# In vitro cellular and proteome assays identify Wnt pathway and CDKN2A-regulated senescence affected in mesenchymal stem cells from mice after a chronic LD gamma irradiation in utero

**DOI:** 10.1007/s00411-021-00925-7

**Published:** 2021-07-21

**Authors:** Martina Schuster, Gargi Tewary, Xuanwen Bao, Prabal Subedi, Stefanie M. Hauck, Ann Karin Olsen, Dag Markus Eide, Klaus Rüdiger Trott, Sebastian Götz, Michael J. Atkinson, Michael Rosemann

**Affiliations:** 1 Institute of Radiation Biology, Helmholtz Zentrum München GmbH, German Research Center for Environmental Health GmbH (HMGU), Ingolstaedter Landstraße 1, 85764 Neuherberg, Germany; 2 Research Unit Protein Science, Helmholtz Zentrum München GmbH, German Research Center for Environmental Health GmbH (HMGU), 80939 Munich, Germany; 3 Department of Molecular Biology/Domain for Infection Control and Environmental Health, Norwegian Institute of Public Health, Lovisenberggt. 8, 0456 Oslo, Norway; 4 Chair of Radiation Biology, Technical University Munich (TUM), 80333 Munich, Germany; 5 Medical Graduate School, Technical University Munich (TUM), 80333 Munich, Germany

**Keywords:** Prenatal irradiation, Low dose irradiation, Mesenchymal stem cells, Senescence, DNA repair, Proteomics

## Abstract

Reliable data on the effects of chronic prenatal exposure to low dose (LD) of ionizing radiation in humans are missing. There are concerns about adverse long-term effects that may persist throughout postnatal life of the offspring. Due to their slow cell cycle kinetics and life-long residence time in the organism, mesenchymal stem cells (MSCs) are more susceptible to low level genotoxic stress caused by extrinsic multiple LD events. The aim of this study was to investigate the effect of chronic, prenatal LD gamma irradiation to the biology of MSCs later in life. C3H mice were exposed in utero to chronic prenatal irradiation of 10 mGy/day over a period of 3 weeks. Two years later, MSCs were isolated from the bone marrow and analyzed in vitro for their radiosensitivity, for cellular senescence and for DNA double-strand break recognition after a second acute gamma-irradiation. In addition to these cellular assays, changes in protein expression were measured using HPLC–MS/MS and dysregulated molecular signaling pathways identified using bioinformatics. We observed radiation-induced proteomic changes in MSCs from the offspring of in utero irradiated mice (leading to ~ 9.4% of all detected proteins being either up- or downregulated) as compared to non-irradiated controls. The proteomic changes map to regulation pathways involved in the extracellular matrix, the response to oxidative stress, and the Wnt signaling pathway. In addition, chronic prenatal LD irradiation lead to an increased rate of in vitro radiation-induced senescence later in life and to an increased number of residual DNA double-strand breaks after 4 Gy irradiation, indicating a remarkable interaction of in vivo radiation in combination with a second acute dose of in vitro radiation. This study provides the first insight into a molecular mechanism of persistent MSC damage response by ionizing radiation exposure during prenatal time and will help to predict therapeutic safety and efficacy with respect to a clinical application of stem cells.

**Supplementary Information:**

The online version contains supplementary material available at 10.1007/s00411-021-00925-7.

## Introduction

Mesenchymal stem cells (MSCs) are adult stem cells in vertebrates which serve as a source for the regeneration of various tissue types throughout the lifetime of an organism. They are a key cell type for repair and regeneration of bone (Jaiswal et al. 1997), cartilage (Johnstone et al. 1998) and adipose tissue (Purpura et al. 2004), but they are also able to give rise to smooth muscle cells (Zhang et al. 2017) and pericytes (Caplan 2017), therefore, playing an important role in vascular function. Apart from being a source for the replacement of lost or worn out cells, MSCs have a secondary function as producer of various cytokines, thereby regulating inflammatory reactions in tissue (Squillaro et al. 2016). Functional impairment of MSCs associated with cellular ageing and senescence has been linked with degenerative diseases, but MSCs are also prone to malignant transformation (Ganguly et al. 2017), with the potential consequence of sarcoma development (Lin et al. 2009; Shimizu et al. 2010).

Due to their slow cell cycle kinetics and their long residence time in the organism (Nombela-Arrieta et al. 2011), MSCs can be affected by a chronic LDR or multiple protracted radiation exposures to a higher degree than cells with a rapid turnover. When DNA damage becomes too extensive due to reduced repair capacity, the cells accumulate irreversible damage, causing either senescence or apoptosis (Alessio et al. 2015; Watters 1999), or they may suffer from mutations and undergo malignant transformation (Rando 2006).

Major results on prenatal radiation exposure in mice were obtained on the basis of three critical prenatal developmental stages, which differ drastically in their consequences in terms of radiation exposure. While no observable effects were found at low doses in the pre-implantation phase, which takes place within the first 5 days of mice development (Jacquet 2004), during organogenesis (days 8–15) and fetal development (days 14–20), the effects are more likely to be associated with behavioral changes and impairment in learning and memory of the irradiated offspring (Gao and Zhou 1998). However, persistent change into adulthood was observed only at higher radiation doses (0.50 or 1.0 Gy) (Minamisawa and Hirokaga 1995; Sreetharan et al. 2017). In mice and rats, LD irradiation exposure of 0.05–0.3 Gy was found to reduce birth weight and growth due to potential stress in utero during development (Devi 1998; Hande et al. 1990; Jensh et al. 1986).

In a first attempt to better understand the influence of a chronic LD irradiation exposure of the developing organism on the regenerative capacity of adult stem cells later in life, C3H/Eyl mice were exposed to prenatal chronic low dose radiation of 10 mGy/day over 3 weeks, resulting in a cumulative total dose of 0.2 Gy. Persisting long-term effects in MSCs were studied in cells collected from adult mice 2 years after fetal irradiation and used for proteome pathway analysis and in vitro cellular experiments.

The results of this study will shed light on the consequences of a low-dose irradiation during embryogenesis with the special focus on the effects onto adult MSCs.

## Material and methods

### Animal breeding and maintenance

Pregnant C3H/Eyl mice were derived from a F1 intercross C3H/H-Eyl/ + at the HMGU breeding facility and shipped overnight to the Norwegian Research Center for Public Health (FHI Oslo). The Eyl-mutant line carries a point mutation in the Pitx3-gene and was part of another study, focusing on late effect in the midbrain. From the mating of the F1 × F1 parental mice, only the Pitx3-wildtype offspring were selected for the study presented here. C3H/H wt mice among the F2 litters were selected by PCR based genotyping from tail tip-DNA. Time point of mating was determined by monitoring formation of vaginal plugs and the pregnant mice were transferred to the FIGARO low-dose rate gamma radiation facility. Eight weeks after birth, offspring mice were transferred back to the Helmholtz Center Munich and kept under SPF conditions with controlled temperature and humidity. The handling and treatment of mice was performed in conformity with the laws and regulations for animal experiments in Norway and were approved by the Norwegian Animal Research Authority. Mice were always fed ad-libitum to Altromin chow and fresh water constantly provided.

### Chronic low dose gamma irradiation

The FIGARO radiation facility consists of a 12 Ci ^60^Co source that provides a gamma dose rate ranging from 3 Gy/h (at source) down to 400 μGy/h, depending on the distance. The mouse cages were placed in IVC racks at a distance from the source of 18 m. At this position, the radiation field was relatively uniform across the cages and had a dose-rate of 0.4 mGy/h. Gamma dosimetry was done using 10 l ionization chambers, and the measured air-kerma rate was converted to a tissue-equivalent dose using a conversion factor of 0.915. The field homogeneity was determined with the help of chemical alanine dosimeters and EPR. Room temperature was 21 °C, 50% humidity, automatic day-night illumination cycle and 300 m^3^/h airflow. Radiation exposure to ^60^Co gamma rays was done for 3 weeks of pregnancy, yielding a cumulative dose of 0.2 Gy.

### In vitro culture of primary murine MSCs

Bone marrow MSCs were isolated from the femur and tibia of prenatally exposed and control male mice at the age of 2 years. Per group, four mice were used and from each animal three independent lines of MSCs were established (from left femur, right femur, and from both tibia). Limbs were cleaned from adherent tissue and bone marrow cells were collected by flushing the marrow cavity with ice-cold PBS, using a 0.4 mm injection needle. The collected cell suspension was homogenized by pipetting it up and down, centrifuged (5 min at 300 g) and the pellet washed again with PBS. Finally, cells were resuspended in DMEM/F12 media containing 10% mesenchymal stem cell qualified FBS (Life Technologies, Carlsbad, CA) and 10 μM rock-inhibitor Y-27632 (Tocris, Wiesbaden, Germany) and plated in wells of 6 well tissue culture plates (Greiner CELLSTAR, Germany) under reduced oxygen (2% O_2_, 5% CO_2_) at 37 °C in a humidified atmosphere. Twice a day over the following 3 days, the non-adherent cells were removed by an exchange of medium. After 7 days and reaching 80% confluency, cells were detached by StemPro Accutase Cell Dissociation Reagent (Life Technologies) for passaging. Cell numbers were determined with a Z1 Coulter Counter (Beckman Coulter, Brea, CA) and cells passaged once a week.

### In vitro gamma irradiation of cultured mesenchymal stem cells

In vitro gamma irradiation was performed using a closed cabinet HWM-D-2000 (Hans Wälischmiller Engineering, Markdorf, Germany) ^137^Cs source providing a dose rate of 0.5 Gy/min. Cells were kept at room temperature during irradiation and control cells were sham-irradiated.

### Clonogenic assay

MSC cells (passage 3) were resuspended in 1 ml PBS and irradiated with 0.5, 1, 2, 4 Gy doses. Controls were sham-irradiated. Cells were plated in 10cm tissue culture dishes (Greiner CELLSTAR, Germany) and cultured following our standard protocol (see above). After 10 days of culture, cells were methanol fixed, colonies stained with crystal violet and counted by eye under 4 × magnification.

Relative Plating efficiency was calculated using following formula:

$${\text{PE}}\left( r \right) = \frac{{{\text{Number}}\,{\text{of}}\,{\text{colonies}}\,{\text{formed}}}}{{{\text{Relative}}\,{\text{cell}}\,{\text{concentration}}}}.$$

The relative cell concentration is presented as the fold cell number for each dose.

Survival Fraction (SF) at a specific dose was calculated using following formula:

$${\text{SF}} = \frac{{{\text{Relative}}\,{\text{plating}}\,{\text{efficiency}}\,{\text{of}}\,{\text{treated}}\,{\text{samples}}}}{{{\text{Plating}}\,{\text{efficiency}}\,{\text{of}}\,{\text{the}}\,{\text{control}}}}.$$

Dose-dependent change of the natural logarithm of SF was fitted to a linear-quadratic model:

$${\text{Ln}}\left( {{\text{SF}}} \right) = - \alpha *D - \beta *D^{2} .$$

### Senescence-associated β-galactosidase activity

To detect the activity of β-galactosidase MSCs (passage 3) were cultured in 24-well plates until they reached 80% confluence. Following washing steps (2 × PBS), cells were fixed with Roth HistoFix (4% PFA, Roth, Karlsruhe) for 10 min, followed by additional washing with PBS. Then, cells were stained with freshly prepared X-gal staining solution [1 mM X-gal; 40 mM Di-Sodium hydrogen-phosphate Di-hydrate; 150 mM NaCl; 2 mM MgCl; 5 mM Potassium-Hexacyanoferrat (II); 5 mM Potassium-Hexacyanoferrat (III)] for 12 h at 37 °C and normal atmosphere. After staining, cells were washed with PBS and cells visualized under bright-field illumination and 10 × lens (Zeiss Axiovert 25 microscope). SA-β-galactosidase positive cells were detected by their turquoise stain and counted relative to the total cell number as determined by phase-contrast.

### Immunofluorescence staining for DNA-repair foci

Cells (passage 3) were cultured overnight on sterile microscope slides (75 by 26 mm) and irradiated with doses of 1, 2 and 4 Gy. 90 min after irradiation, cells were fixed with Roth HistoFix for 10 min followed by two washes with PBS. Cells were permeabilized with 0.2% Triton X100 in PBS for 1 h, blocked for 1 h in 1% BSA, 0.15% glycine in PBS then incubated overnight with a combination of mouse monoclonal anti-γH2AX antibody (Merck Millipore, Schwalbach, 1:500) and a rabbit polyclonal anti-53BP1 antibody (Novus Biological, Littleton USA, 1:500) at 4 °C. Following extensive washing steps (3 × 15 min in PBS/1% BSA), secondary antibodies (1:500 Cy3-conjugated sheep anti-mouse IgG and 1:200 Alexa 488-conjugated goat anti-rabbit IgG, Jackson Immunoresearch, West Grove) diluted in PBS/1% BSA were applied to the cells for 1 h at room temperature. Finally, additional washing steps were performed as described above, cells counterstained with Hoechst 33,342 solution (Life Technologies) and mounted with Vectashield (VectorLabs, Burlingame, Ca).

Images of γH2AX and 53BP1 foci were taken with a fluorescent microscope (BZ-9000; Keyence, Osaka, Japan) and associated BZ II viewer software. γH2AX and 53BP1 were detected by excitation of the Cy3 and Alexa488 laser and then visualized by the 100 × oil immersion lens. At least 100 nuclei were analyzed. For the dose–response of radiation-induced DNA double-strand breaks the mean number of foci and the standard error of mean of counted foci were plotted in a linear mode vs. the in vitro radiation dose.

### Statistical analysis

Statistical analysis was performed using two-way ANOVA (GraphPad5, GraphPad Software, San Diego, CA, USA) with the *p*-value ≤ 0.05. All results are given as mean values with their standard errors (SEM). Four biological replicates were recorded for proteomic analysis and three for the in vitro analysis (Induction of DNA repair foci, induction of cellular senescence, clonogenic assay). Replicates were MSCs derived from separate in utero irradiated mice and compared with MSCs of the same number of sham irradiated control mice.

### Protein lysis and measurement of protein concentration

MSC pellets (pooled from three independent MSC cultures per animal) were homogenized and lysed in RIPA buffer (Thermo Fischer, Darmstadt, Germany) containing phosphatase and protease inhibitors (Sigma-Aldrich, Taufkirchen, Germany). After sonication and lysis, the protein concentrations were determined with the BCA Protein Assay (Thermo Fischer) according to the manufacturer’s instructions. The protein lysates were stored at − 80 °C. 10 µg of lysate per sample were used for LC-MSMS analysis.

### Mass spectrometry

Proteins extracted from MSC pellets (10 µg) were proteolysed with Lys-C and trypsin by a filter-aided sample preparations method as previously described (Grosche et al. 2016). Eluted peptides were supplied with indexed retention time (iRT) hyper reaction monitoring (HRM) calibration peptides (BIOGNOSYS, Schlieren, Switzerland) (Bruderer et al. 2016) for retention time alignment and then analyzed in data-independent acquisition (DIA) mode on a Q Exactive high field (HF) mass spectrometer (MS) (Thermo Fisher Scientific, Waltham, MA, USA) as described (Lepper et al. 2018). Briefly, MS spectra were recorded from 300 to 1650 m/z at 120,000 resolution with an automatic gain control (AGC) target of 3e6 and a maximum injection time of 120 ms. Data-independent acquisition windows were selected based on previous data-dependent acquisition runs, with a scan resolution of 30,000, an AGC target of 3e6 and a collision energy of 27.

### Protein identification and quantification

The recorded raw-files were analyzed using the Spectronaut software (BIOGNOSYS, version 12) (Bruderer et al. 2015) as described (Lepper et al. 2018) with an in-house database spectral library which was generated using Proteome Discoverer 2.1, Mascot search engine (Matrix Science, London, UK) and the Swissprot Mouse database (release 2016_02). Quantification was based on cumulative MS2 area levels with the *q*-value percentile 0.2 setting. Proteins identified and quantified with two UP, *q*-value of ≤ 0.01 and fold-changes of ≤ 0.77 or ≥ 1.3 were considered to be significantly differentially expressed.

### Bioinformatic analysis

To create a functional profile of all significantly deregulated proteins, the Gene Set Enrichment Analysis (GSEA) was performed with the GSEA software of Broad Institute. Signaling networks, protein–protein interactions and the analysis of upstream regulation in response to irradiation were identified by uploading the list of deregulated proteins with their accession numbers to the STRING protein database (http://string-db.org), to Metascape (http://metascape.org/), and to Ingenuity Pathway Analysis (IPA) software (QIAGEN Redwood City, http://qiagen.com/ingenuity). The proteins were filtered in advance according to preset criteria (*q*-value ≤ 0.01 and fold-change |LogFC|> 1.3).

## Results

### Chronic low-dose radiation in utero induces changes in the proteome of MSCs in adult mice

**Fig. 1:**
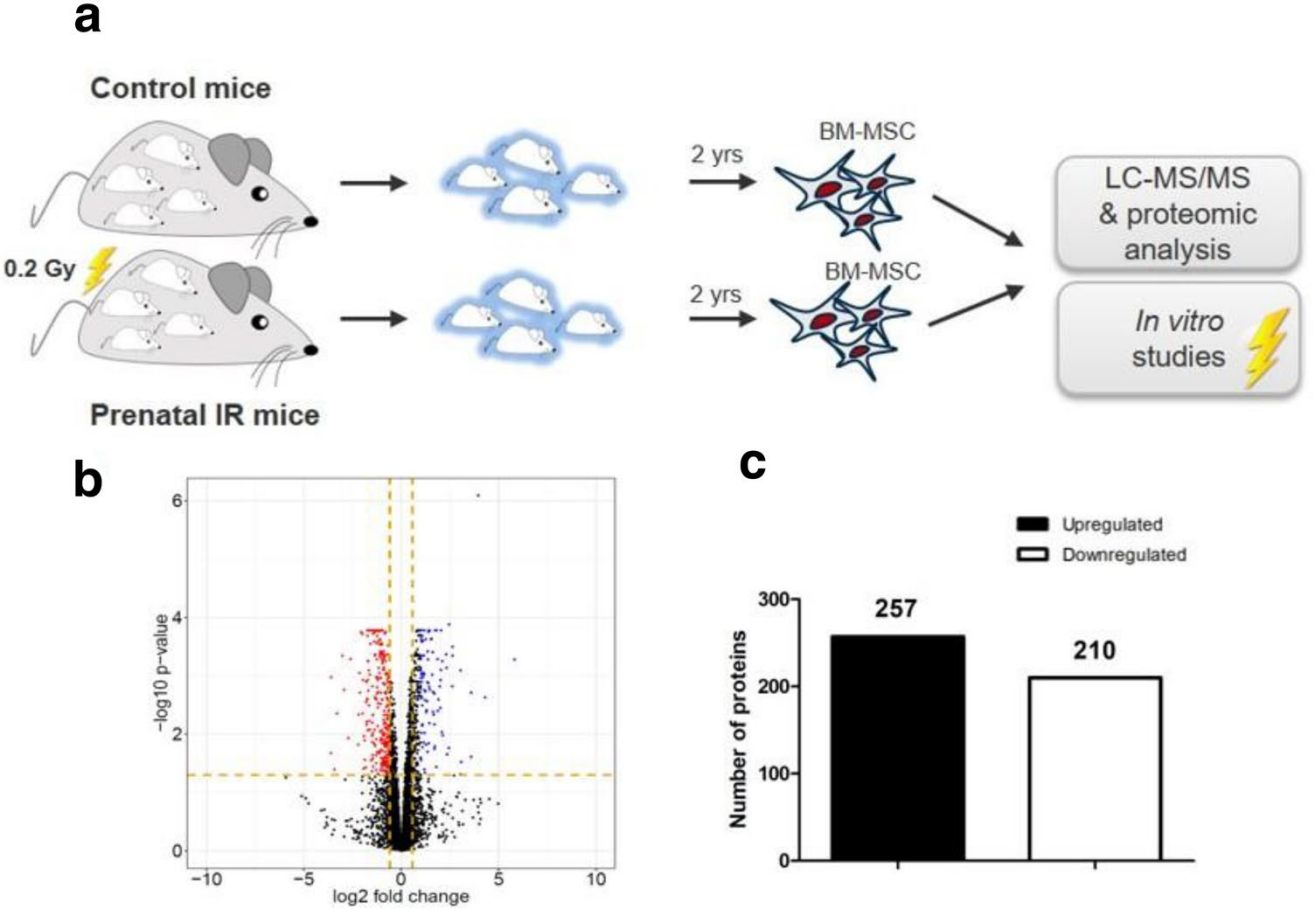
Chronic low dose radiation effects on the proteome of mesenchymal stem cells. **a** Workflow and experimental design of the study. Control mice (non-irradiated) and prenatal irradiated mice (exposed group) are shown. **b** Volcano plots representing the distribution of all quantified proteins (identification with ≥ 2 peptides) in mesenchymal stem cells exposed to a chronic radiation dose in the uterus of in total 0.2 Gy compared to non-irradiated controls. Upregulated proteins are shown in red and downregulated in blue, fold change ± 1.3. **c** The total numbers of significantly upregulated and downregulated proteins are shown for all dose groups (*q* ≤ 0.01, fold change ± 1.3)

The adult mice that were used as MSC donors had received in utero a chronic gamma irradiation with a cumulative dose of 0.2 Gy. The radiation lasted from the time point of conception until birth, therefore, covering both the embryonal as well as the fetal developmental stage. At the age of 2 years, these mice were sacrificed and short-term MSC cultures establish from their bone marrow. The proteome was analyzed following expansion of these MSCs in vitro over a time of 3 weeks (Fig. 1a).

In all samples of MSCs, a total of 4987 proteins could be identified unambiguously and quantified for their relative change in expression level. Using a cut-off of absolute fold change higher than 1.3 and significance of difference *q* ≤ 0.01, 467 of all proteins were dysregulated in their expression level when MSCs were derived from prenatally irradiated mice (Fig. 1b). In the volcano plot 257 proteins (or 55.0%) were found upregulated and 210 proteins (or 45%) downregulated (Fig. 1c). Unsupervised clustering of dysregulated proteins in one dimension and sample ID in the second dimension shows a consistent separation between MSCs from control mice and from mice which received an in utero LD gamma irradiation (Fig. S1).

### Prenatal chronic low dose in utero irradiation leads to induction of radiation induced senescence later in life

**Fig. 2:**
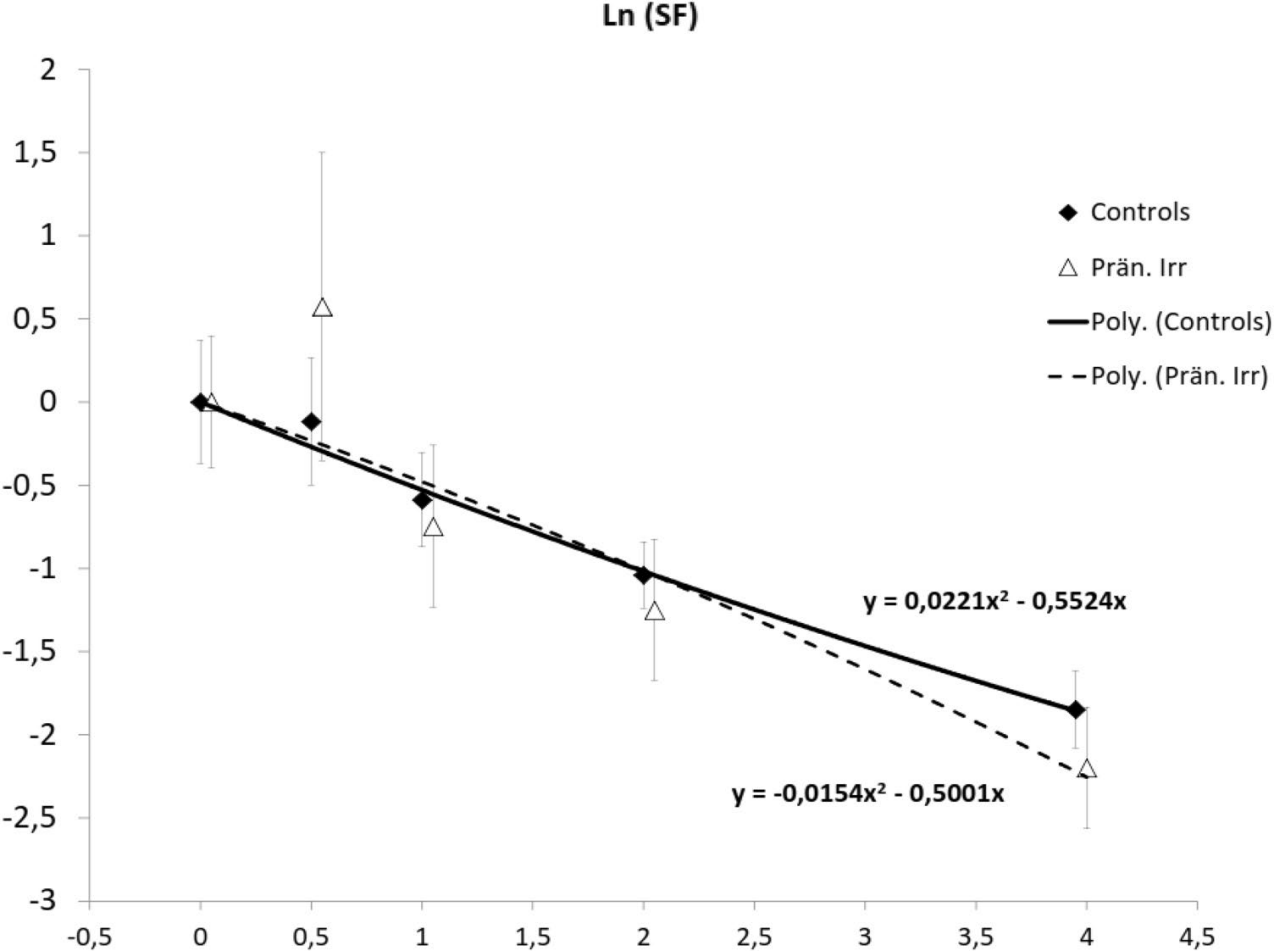
Clonogenic survival of in vitro gamma irradiated MSCs derived from in utero exposed or control mice. Error bars represent the SEM. Lines are linear-quadratic fits of the natural logarithm of the survival ration. Equations give the alpha and beta values of the fitted curves

The clonogenic survival potential of MSCs was not significantly altered following prenatal in vivo exposure of mice compared to non-irradiated controls (Fig. 2). Within the limits of precision of the assay, the Ln(survival-fraction) was following almost entirely a linear dependence on dose in the range between 0 and 4 Gy, with alpha-values of 0.55 (CI 0.51–0.59) for the control MSCs and 0.5 (CI 0.44–0.56) for MSCs after in utero irradiation.

**Fig. 3:**
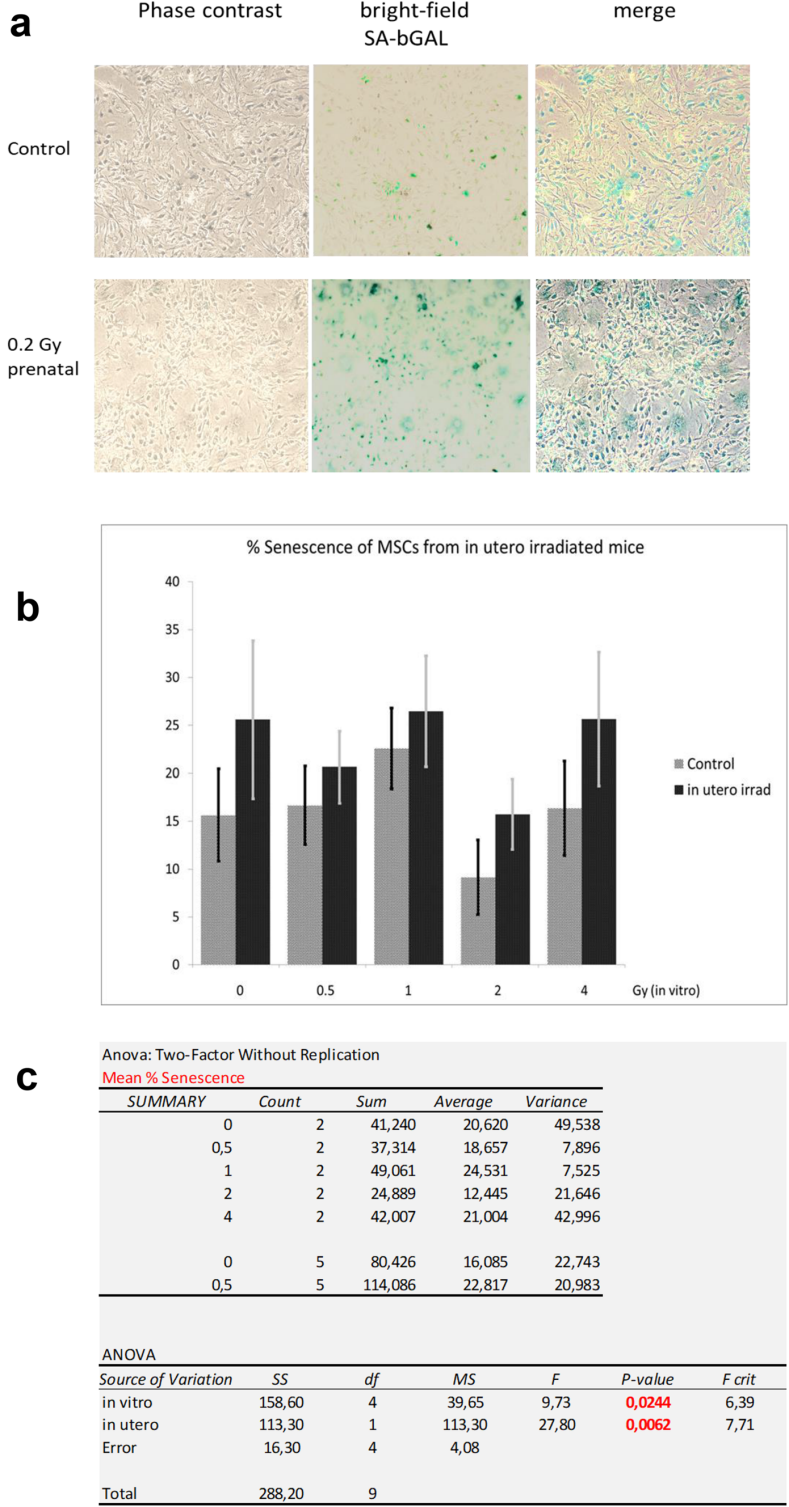
Induction of senescence in MSCs of in utero irradiated and non-irradiated mice. **a** Images of SA-βGal stained MSCs in vitro by phase contrast (left column), bright field (middle column) and overlay (right column). **b** Percentage of cells undergoing senescence, mean values and SEM, *n* = 3. **c** Results of two-way ANOVA analysis showing highly significant influence of in utero LD gamma irradiation onto MSC senescence

The percentage of senescent cells in MSCs in vitro displayed considerable variation, both depending on the in utero irradiation status as well as following a second irradiation of the MSCs in vitro. MSCs derived from in utero non-irradiated mice contain between 9.2% and 20.2% senescent cells (fluctuating with the level of additional in vitro radiation), as compared to a range of 15.7–26.5% senescent cells within MSCs from mice irradiated in utero with 0.2 Gy. The additional in vitro irradiation with doses from 0.5 Gy to 4 Gy did not cause a clear dose-dependent rise in the fraction of senescent cells, rather than fluctuations around the control level. Averaged over all in vitro doses, MSCs derived from mice after in utero irradiation had a 1.45-fold increased level (CI 1.38–1.85) in the fraction of senescent cells as compared to MSCs that were established from unirradiated mice (*p* = 0.006, two-way ANOVA) (Fig. 3). This suggests that in vivo prenatal irradiation of mice has a significant effect on the radiation-induced cellular senescence in MSCs later in life.

### Induction of DNA repair foci in adult MSCs derived from in utero irradiated mice is increased

**Fig. 4:**
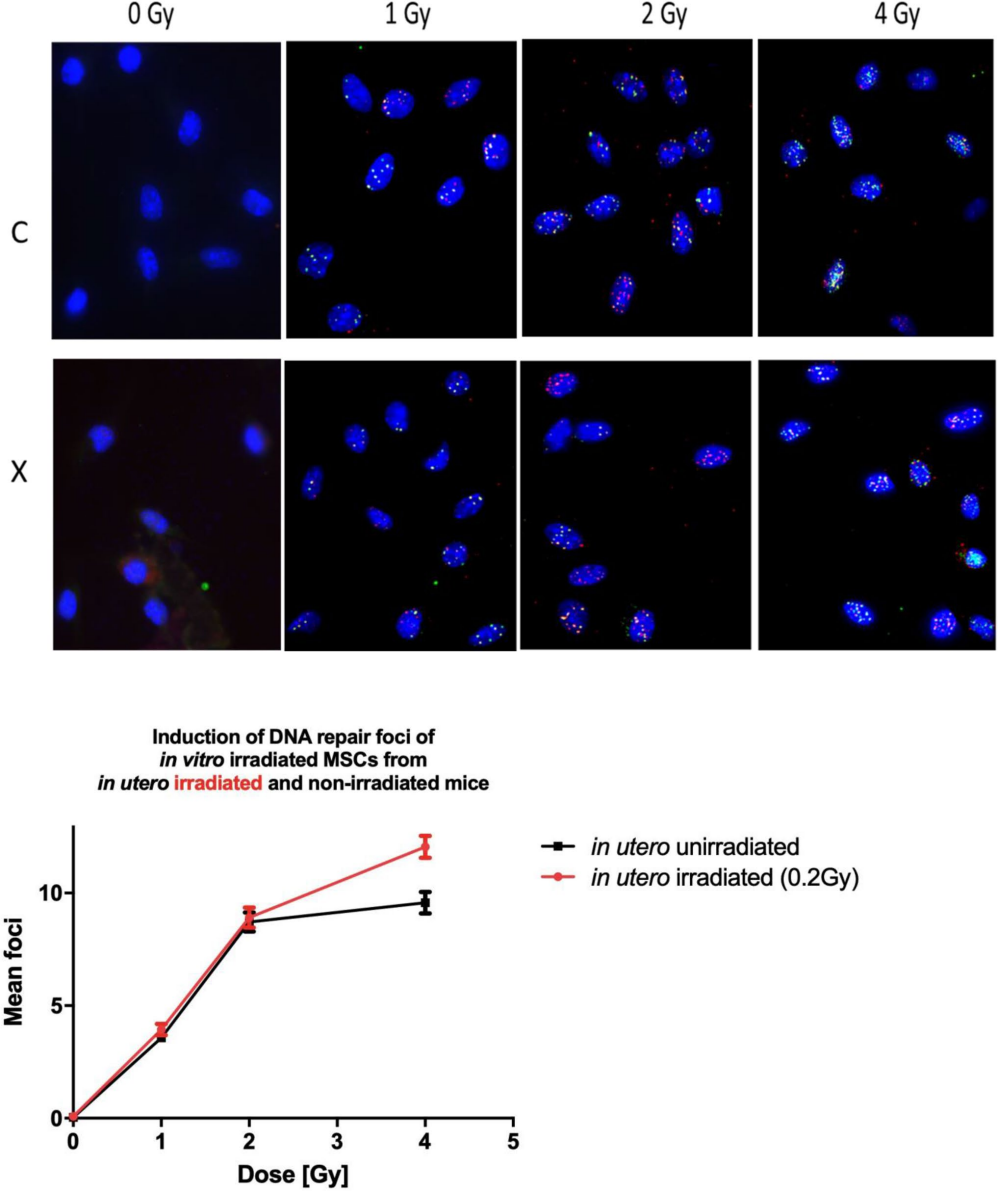
Induction of DNA repair foci in MSCs derived from in utero gamma exposed or control mice following an additional in vitro irradiation of the cells. Average of co-localized γH2AX- and 53BP1-foci per MSC 90 min after their in vitro irradiation with increasing radiation doses. Error bars represent the SEM, *n* = 3, Higher foci number at 4 Gy in in utero gamma exposed MSCs is significant at *p* = 0.001, (two-way ANOVA)

We observed that 90 min after an acute gamma irradiation in vitro, cells from both groups of mice displayed a steady rise in foci number with dose by between 1 and 4 Gy. Only at the highest in vitro dose of 4 Gy, in MSCs from in utero irradiated mice a small but significant increase of γH2AX/53BP1 foci was seen compared to cells from in utero non-irradiated mice. In particular, there was also no indication of a higher basal frequency of γH2AX/53BP1 foci in cells derived from prenatally exposed as compared to control mice, indicating spontaneous DNA double-strand breaks are not increased 2 years after in utero irradiation. In MSCs from in utero irradiated mice the mean foci number after 4 Gy was 1.28-fold higher than in the MSCs from in utero non-irradiated mice (Fig. 4). Although this increase was clearly significant (**p = 0.0015), considering that the effect is relatively small and only found at the highest dose point one cannot exclude the possibility that a confounding factor (for instance cell cycle arrest or changes on chromatin architecture) underlies this difference.

### Bioinformatic characterization of the functional groups of deregulated proteins

**Fig. 5:**
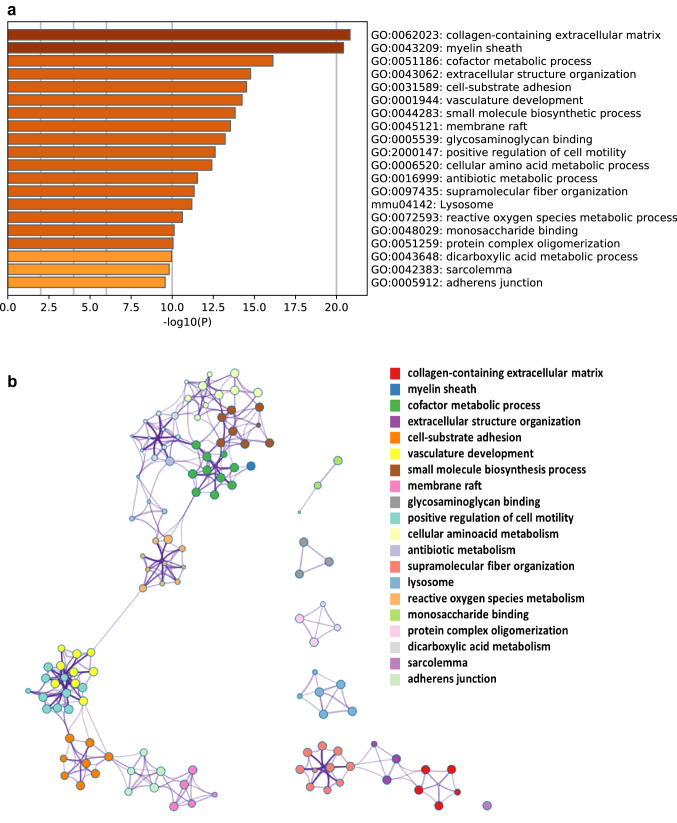
Metascape analysis of significantly deregulated proteins of MSCs from prenatally irradiated mice. **a** Top 20 clusters of GO enrichment analysis are shown with **b** top 20 clusters represented as a network

**Fig. 6:**
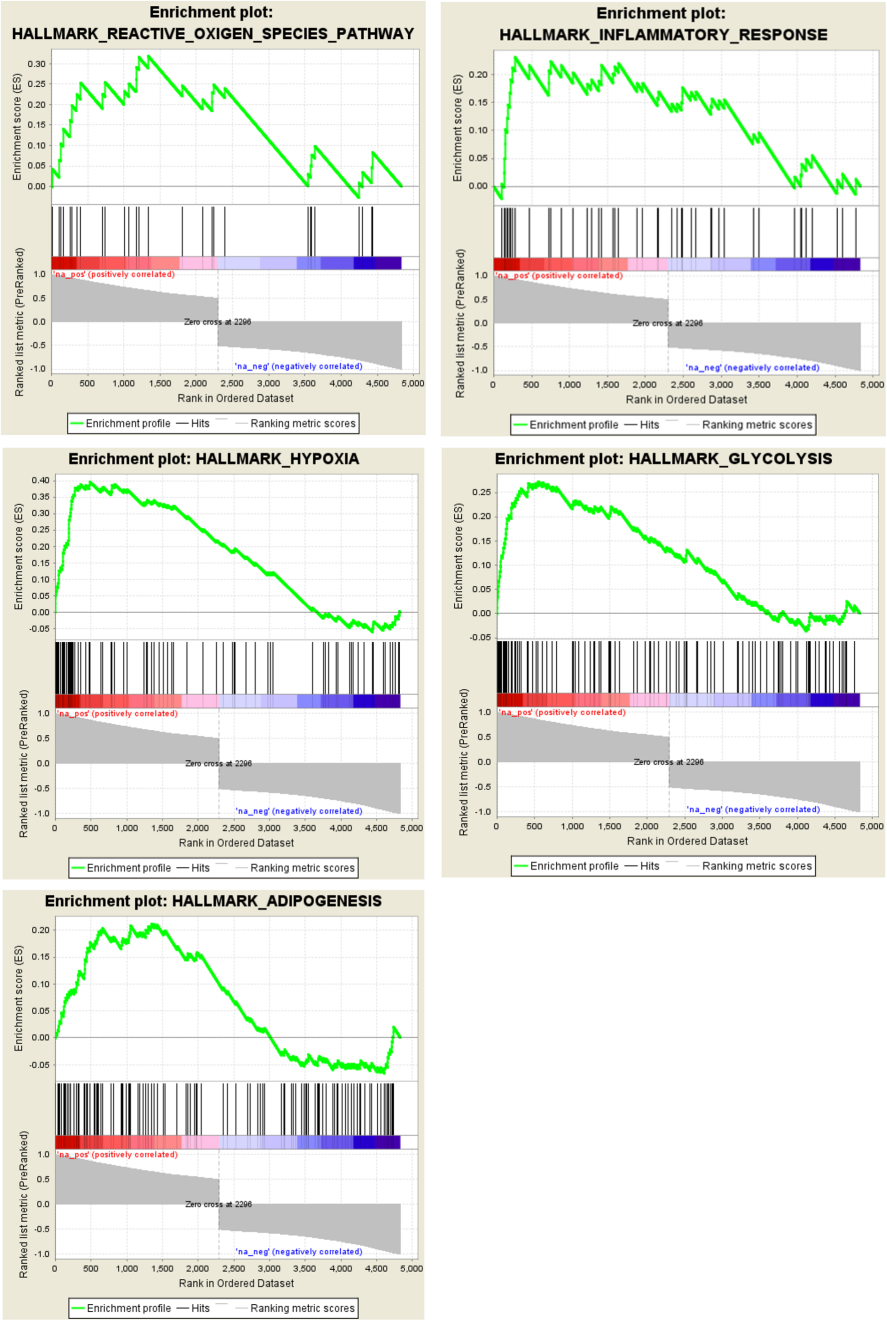
Gene set enrichment analysis (GSEA) of significantly deregulated proteins of MSCs from prenatally irradiated mice. The five most significantly enriched gene sets are shown

Gene ontology (GO) analysis revealed that proteins in MSCs affected by a preceding in utero gamma irradiation were associated with altered metabolic pathways (GO: 0,051,186), including detoxification of reactive oxygen (GO: 0,072,593), cell adhesion (GO: 0,031,589) and modifications of the collagen-containing extracellular matrix (GO: 0,062,023). The top 20 were shown in Fig. 5a and represented as a network analyzed by Metascape (Fig. 5b). To reduce the complexity of the list and to identify specific proteins, gene set enrichment analysis (GSEA) was performed. Among the five most significantly enriched gene sets are the hallmarks reactive oxygen species, hypoxia, glycolysis, adipogenesis and inflammatory response (Fig. 6).

**Fig. 7:**
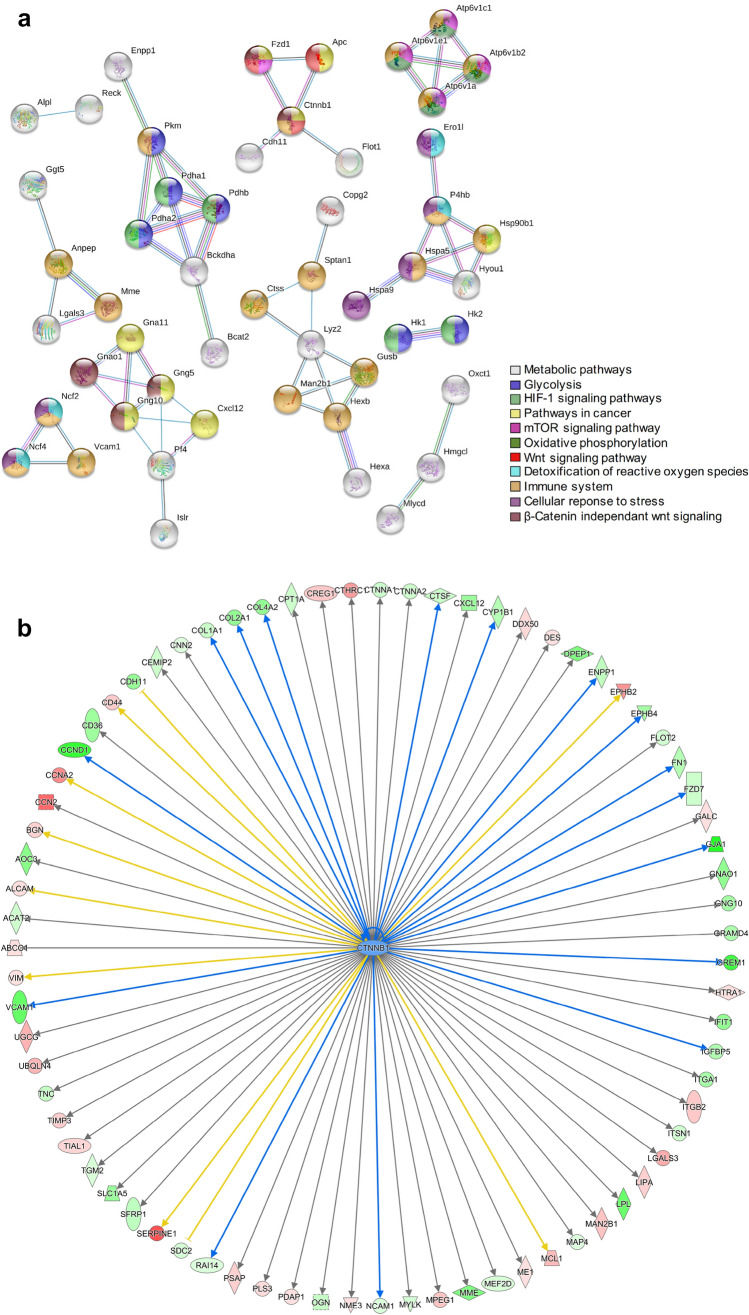
Molecular pathways affected in in utero irradiated MSCs. MSCs from in utero exposed mice exhibit changes in gene networks related to metabolic pathways, immune system, hypoxia and Wnt-pathway. **a** The 100 most significant deregulated proteins (*q* ≤ 0.01, fold change ± 1.3) were used for STRING functional protein interaction analysis. **b** Analysis of predicted upstream regulators using IPA. Graphical presentation of deregulated proteins with their upstream regulator beta-catenin (CTNNB1) in the irradiated cells is shown. Functional downregulation of CTTNB1 (indicated by the blue color) would be associated with a Wnt-pathway inactivation

To gain more biological insight, an interactome of the 100 most significant deregulated proteins was created using the STRING-db software. The network analysis supports the involvement of metabolic pathways, immune system and hypoxia and indicates that the Wnt-pathway also is altered (Fig. 7a).

A prediction of possible upstream regulators of altered proteins was performed (IPA). Ctnnb1 (Fig. 7b) and Sirt1 (Fig.S2) were predicted as inactivated, while Cdkn2a and HIF1 (Fig.S2) to be activated in adult MSCs derived from mice prenatally exposed.

## Discussion

In this study, we have shown that chronic low dose exposures to IR (in total 0.2 Gy) in the uterus, comparable to those received by repeated CT scans, had an effect on the proteome of murine mesenchymal stem cells two years later. There is a gap in knowledge about the long-term damage that can be caused by chronic low dose prenatal exposure. Interestingly, Howell et al. have found that low-dose exposure of mice during pregnancy (0.13 Gy over 10 days) was not directly harmful to the offspring, but suggest a radio-adaptive response in damage response, blood cell levels and expression of genotoxic pathway genes following a second acute high dose exposure in the newborn (Howell et al. 2013). This may be consistent with an increased efficiency of DNA repair, impaired immune response and oxidative stress metabolism, as well as the increased possibility of apoptosis to remove damaged tissue (Feinendegen 2005; Ikushima et al. 1996). In their study, however, the second acute radiation treatment of the prenatally exposed mice occurred 16–18 days after the initial exposure. The long-term maintenance of such a protective mechanism after exposure is unclear, especially regarding the consequences of low-dose in-utero radiation on the bone marrow and its stem cells (MSCs). Severe health effects on the neonate after in utero exposure of > 0.1 Gy were frequently observed after the Chernobyl accident and atomic bomb attacks on Hiroshima and Nagasaki, where the newborn suffered from down syndrome, neural tube defects, microcephaly, small head size and abnormal neuronal migration (Otake and Schull, 1984; Otake et al. 1991; Schull, 1983; Sperling et al. 1991). Migrating neuronal precursors originate from the neural crest during early embryonal neurogenesis. However, MSCs are typical adult stem cells that play a life-long role in immune response, inflammation, tissue regeneration and repair (Squillaro et al. 2016). The observed changes in the proteome profiles could result from slow changes in the differentiation program of these cells, in particular the increased fraction of senescent cells.

In our results, we found 467 deregulated proteins in MSCs 2 years after chronic in utero radiation of mice, indicating a strong radiation effect on the bone marrow and its MSCs. We were then able to show that many highly significant proteins involved in metabolic pathways and the organization of the extracellular matrix (ECM) show different expression 2 years after prenatal exposure. In particular, the expression level of components of the ECM is altered, suggesting that cell migration and adherence capacity of MSCs may be impaired. Low-dose radiation exposure causes cellular stress through the formation of reactive oxygen species, DNA damage and autophagy (Kim et al. 2019). We found an upregulation of proteins involved in the cellular defense of reactive oxygen species, indicating a response to persistent radiation-induced damage even 2 years after in utero exposure. The analysis of signaling pathways also points to a functional increase in hypoxia, glycolysis and adipogenesis, all of which contribute to the radiation response and are associated with increased ROS in cells (Islam et al. 2015; Moeller et al. 2004; Schaue et al. 2015; Zhong et al. 2013).

The Wnt signaling pathway was identified as a strong contributor and verified by IPA analysis as strongly downregulated. Wnt signaling supports tissue stem cell proliferation by stimulating cell division and inhibiting differentiation and apoptosis (Reya and Clevers, 2005). However, we found no significant change in the number of colonies and survival fraction of MSCs from in utero irradiated mice compared to non-irradiated controls. It was already investigated that Wnt/β-Catenin signaling is also responsible for the aging of MSCs through the involvement of DNA damage events (Zhang et al. 2011). We were able to show that prenatal chronic low dose in utero irradiation leads to induction of radiation-induced senescence later in life, suggesting early radiation-induced damage and failure of mitotically active bone marrow stromal cells. These cells carry the potential risk of cellular alterations leading to cellular senescence and probably transformation due to accumulation of DNA damage (Alessio et al. 2015; Rando 2006). It is worth noting that the chronic in utero irradiation (0.2 Gy) increases in vitro senescence in MSCs two years later by 4%—10%, whereas an additional gamma irradiation of the isolated MSCs had no consistent effect onto senescence. The most likely explanation for this is the different time periods between irradiation and manifestation of cellular senescence. Whereas senescent MSCs were counted 24 months after in utero irradiation, but only 3 weeks after in vitro irradiation, the much longer time period following the in utero irradiation could give cellular senescence a significantly longer time for its manifestation. This is consistent with the model of cellular senescence being the limiting factor for long time cell proliferation. Interestingly, we have also shown that DNA repair foci in MSCs from in utero exposed mice are significantly elevated after 4 Gy in vitro irradiation, but it is unclear if this indicates more initial breaks or reduced DNA double-strand break repair.

It has been reported that ectopic expression of cell cycle genes (e.g., p21 and p16) triggers cellular senescence (Chen et al. 2006). In our study, IPA analysis identified that p16 (CDKN2a) is upregulated in MSCs from prenatally irradiated mice. In addition, cellular senescence may also be induced by various stress factors such as oxidative stress (Chen et al. 2007), a factor that is highly upregulated in the MSCs from *in-utero* irradiated mice.

In summary, our study provides the first long-term observation of bone marrow derived MSCs 2 years after a prenatal chronic low-dose exposure to ionizing radiation. We could show that in utero irradiation can induce cellular senescence and alter the efficiency of DNA repair. This could be caused by the accumulation of free radicals or downregulation of the Wnt signaling pathway. We also observed that the expression level of components of the ECM is strongly altered, suggesting that cell migration and adherence capacity of MSCs may be impaired. This, together with the decreased expression of β‐Catenin (Wnt signaling), which has recently been shown to maintain the stemness and stability of MSCs (Sen et al. 2020), indirectly suggests that low doses of radiation in utero, comparable to those obtained from repeated CT scans, may accelerate the normal aging process and alter the beneficial properties of MSCs. Considering that MSCs provide a reservoir for the regeneration of different cell types and an be affected in various diseases, the occurrence of dysregulated, essential signaling pathways even 2 years after prenatal exposure highlights a potential risk of health problems that may occur later in life. This must be taken into account by considering MSCs from donors as possible tools for stem cell therapy to ensure safe and effective treatment.

## Supplementary Information

Below is the link to the electronic supplementary material.

Supplementary file1 (PDF 92 kb)

Supplementary file1 (PDF 12053 kb)

Supplementary file1 (PDF 76 kb)

## Data Availability

Raw proteomics data are available upon request.
