# Correction to: In vitro cellular and proteome assays identify Wnt pathway and CDKN2A-regulated senescence affected in mesenchymal stem cells from mice after a chronic LD gamma irradiation in utero

**DOI:** 10.1007/s00411-021-00945-3

**Published:** 2021-10-02

**Authors:** Martina Schuster, Gargi Tewary, Xuanwen Bao, Prabal Subedi, Stefanie M. Hauck, Ann Karin Olsen, Dag Markus Eide, Klaus Rüdiger Trott, Sebastian Götz, Michael J. Atkinson, Michael Rosemann

**Affiliations:** 1 Institute of Radiation Biology, Helmholtz Zentrum München GmbH, German Research Center for Environmental Health GmbH (HMGU), Ingolstaedter Landstraße 1, 85764 Neuherberg, Germany; 2 Research Unit Protein Science, Helmholtz Zentrum München GmbH, German Research Center for Environmental Health GmbH (HMGU), 80939 Munich, Germany; 3 Department of Molecular Biology/Domain for Infection Control and Environmental Health, Norwegian Institute of Public Health, Lovisenberggt. 8, 0456 Oslo, Norway; 4 Chair of Radiation Biology, Technical University Munich (TUM), 80333 Munich, Germany; 5 Medical Graduate School, Technical University Munich (TUM), 80333 Munich, Germany

## Correction to: Radiation and Environmental Biophysics (2021) 60:397–410 10.1007/s00411-021-00925-7

Authors would like to correct the typo in author name of the co-author. The author name is corrected from "Ann Karin Olson" to "Ann Karin Olsen" in the original publication.

The original article has been corrected.

