# Supplementary figures and images for: In vitro cellular and proteome assays identify Wnt pathway and CDKN2A-regulated senescence affected in mesenchymal stem cells from mice after a chronic LD gamma irradiation in utero
Source: Radiat Environ Biophys. 2021 Jul 21;60(3):397–410. doi: 10.1007/s00411-021-00925-7 (PMC8310520; doi:10.1007/s00411-021-00925-7)

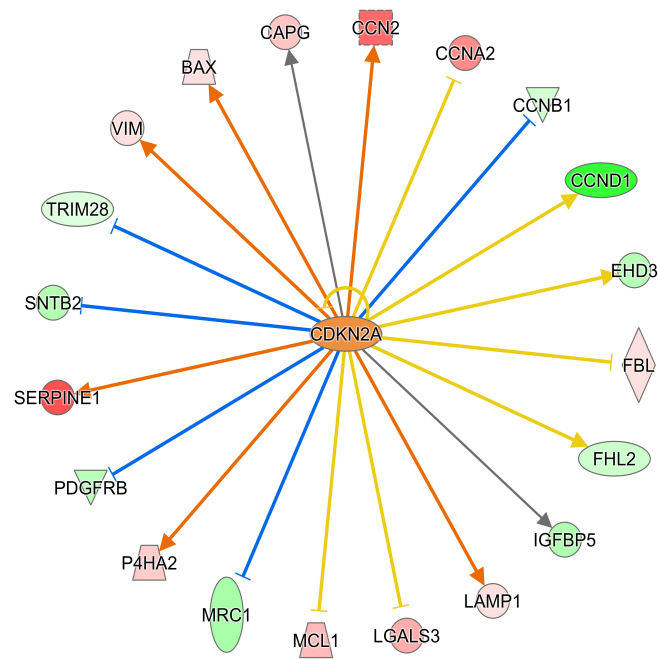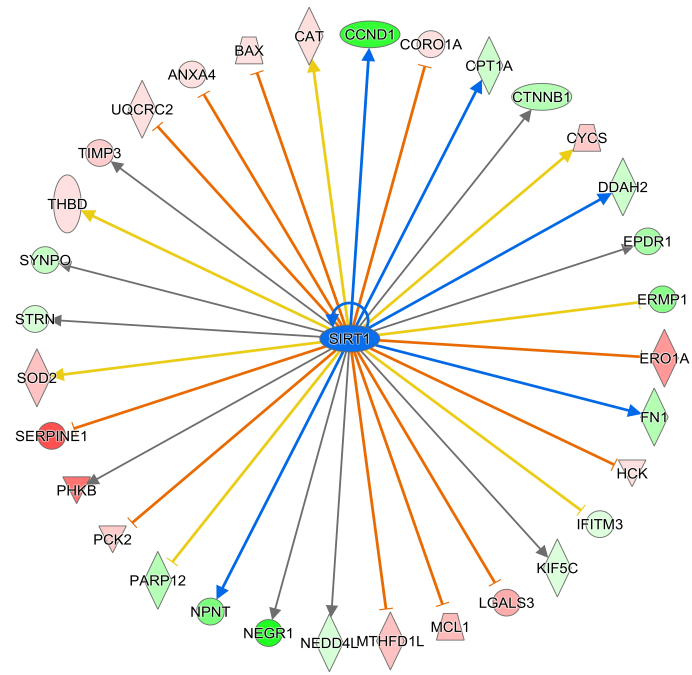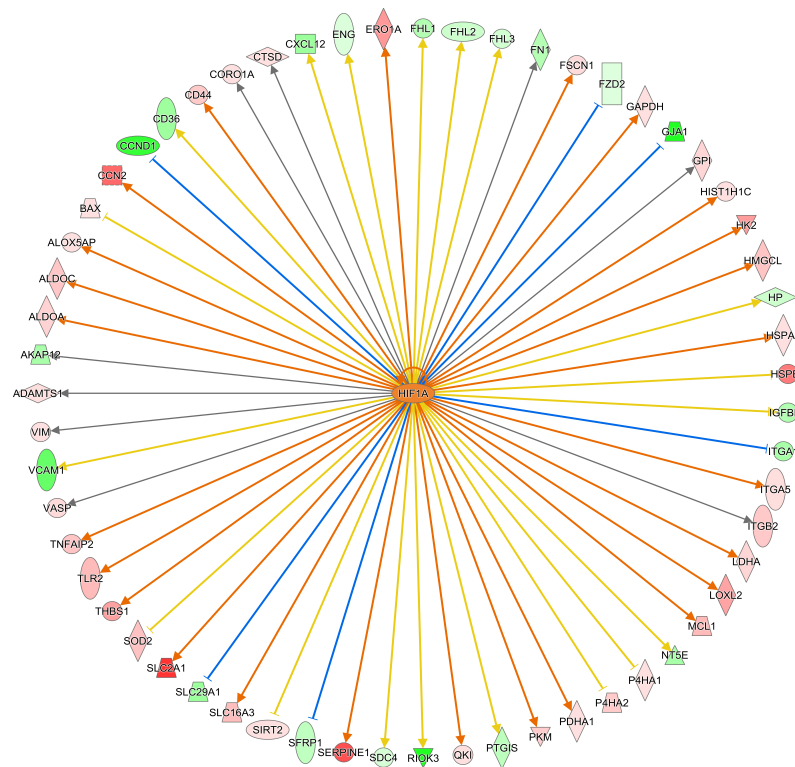

Fig S2

Supplement: Supplementary file 2 — Supplementary file1 (PDF 12053 kb) [file 411_2021_925_MOESM2_ESM.pdf]

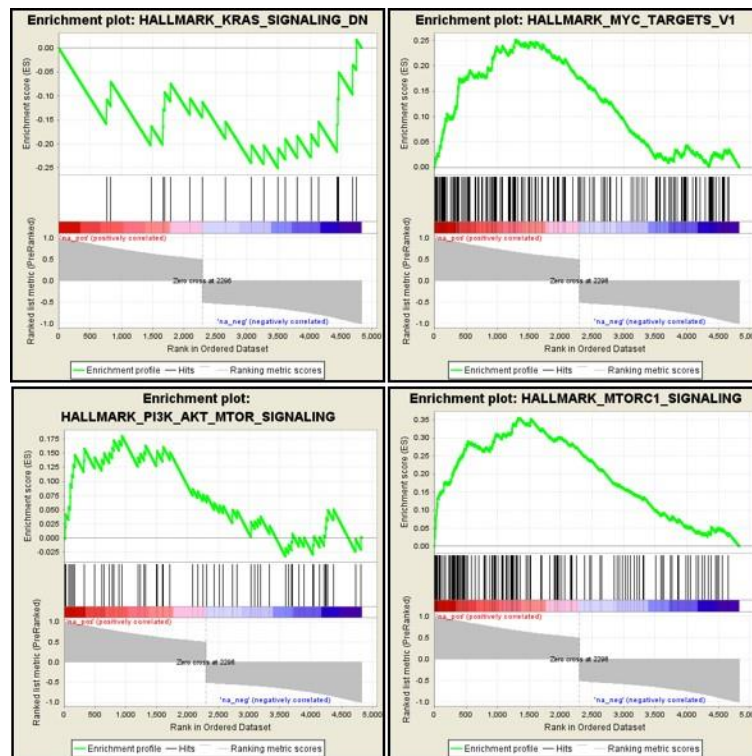

Figure S3

Supplement: Supplementary file 3 — Supplementary file1 (PDF 76 kb) [file 411_2021_925_MOESM3_ESM.pdf]
