# Supplementary material for: In vitro cellular and proteome assays identify Wnt pathway and CDKN2A-regulated senescence affected in mesenchymal stem cells from mice after a chronic LD gamma irradiation in utero
Source: Radiat Environ Biophys. 2021 Jul 21;60(3):397–410. doi: 10.1007/s00411-021-00925-7 (PMC8310520; doi:10.1007/s00411-021-00925-7)
Supplement: Supplementary file 1 — Supplementary file1 (PDF 92 kb) [file 411_2021_925_MOESM1_ESM.pdf]

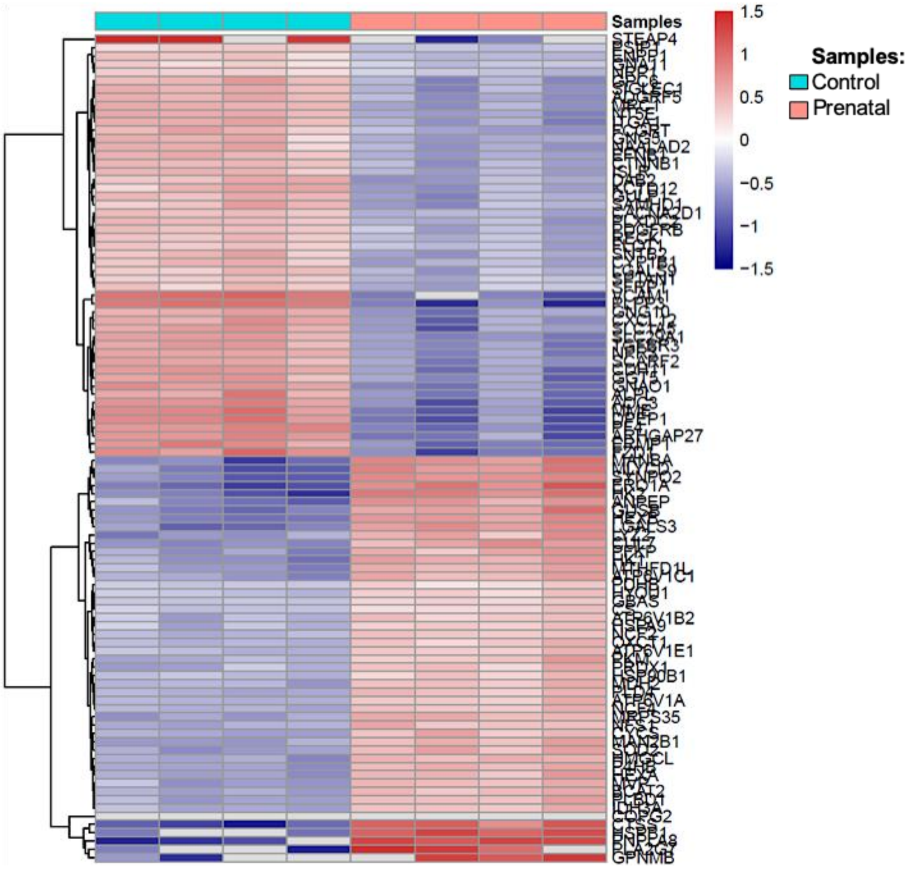
Samples
STEAP4
ARHGAP27
LGALS3
ATP6V1B2
ATP6V1E1
ATP6V1A
MAN2B1
GPNMB
1.5
1
0.5
0
−0.5
−1
−1.5
Samples:
Control
Prenatal

Figure S1
